# Is the Condition Usually Diagnosed Spider Bite Due to the Bite of a Spider?

**Published:** 1895-09

**Authors:** W. T. Richmond

**Affiliations:** Manor, Texas


For the Texas Medical Journal.

### IS THE CONDITION USUALLY DIAGNOSED SPIDER BITE DUE TO THE BITE OF A SPIDER.

BY W. T. RICHMOND, M. D., MANOR, TEXAS.

Read before the Austin District Medical Society, June 20, 1895.

THE condition above referred to makes its appearance as one or more vesicles. If there be several they are crowded close together and eventually coalesce. The patient's attention is first attracted to the place by a sensation of itching. Swelling and redness soon follow, and are accompanied with pain. The swelling and redness extend together and gradually disappear. The redness and pain sometimes follow the course of the lymphatics, and the neighboring lymphatic glands become painful. There is fever and headache; in fact, the patient will say that he aches all over. In about twenty-four hours the vesicles begin to turn dark, and by the end of forty-eight hours may be black. The size of this black spot will vary in different cases, from a quarter of an inch in diameter to the size of a nickel.

Soon after the vesicles make their appearance they rupture and continue to discharge a little fluid. Ulceration begins around the edges of the black spot, which eventually sloughs out, involving the thickness of the skin, and perhaps some of the subcutaneous tissue, leaving a deep ulcer, with ragged and elevated edges. At this time convalescence usually begins.

It will be noticed that the affection begins imperceptibly, gradually increasing in severity for several days before the climax is reached. The venom of insects and serpent is a toxine, and can not increase its destructive power after it is injected under the skin. On the contrary, the resisting powers of the body begin to destroy or eliminate the poison immediately.

I do not know why this condition was ever called spider bite. I have never seen a case of this kind where the patient knew posititvely that a spider had bitten him. In some cases the patient may remember to have seen a spider on his person a day or so before the appearance of the malady, but it made no impression on his mind at the time. The sting of a wasp or the bite of a serpent is very painful from the beginning. Swelling and redness rapidly follow. The effects of the poison are felt and seen immediately.

I was called to see a case of so-called spider bite in a child about two and a half years old. I found the characteristic purple spot in Scarper's triangle. The child's temperature was then about 103. The thigh was swollen on the inner side and quite red, but did not look like erysipelas. I gave quinine internally, and used poultices, antiseptics and anodynes, locally at first. The case grew worse; I used stimulants internally and an ointment of iodol externally. The child lived about ten days from the time it was taken, and died.

A man consulted me about what he called a spider bite on his arm. I found a black spot about half way between the wrist and elbow. The entire fore arm was swollen and red; a red streak extended above the elbow in the direction of the axilla. The axillary glands were painful. He had fever. I told him that the black spot would come out, and I believed it would be best to cut it out. He consented, and I removed it with a knife, and applied carbolic acid to the wound. The inflammation began to subside at once, and he was soon well.

A man engaged in taking up oats that had been piled for some time in a field, was bitten on the shoulder by a spider. The spider was killed and taken from under his shirt. I saw the man an hour afterwards. He told me the pain was instantaneous, and much like the sting of a wasp. There was swelling and redness for some distance around the bite. The man was very nervous and sick at his stomach. His pulse was weak and rapid. I gave a dose of morphine to relieve pain, and aromatic spirits ammonia internally and externally. The next day the man was well.

The difference between the bite known to be that of a spider and the one supposed to be a spider bite is quite distinct.

I believe the affection is due to an inoculation, probably by a fly that has been feeding on a putrescent or diseased body, possibly on an animal that has died of anthrax.

A fly could carry to the wound a germ, which would reproduce itself, and produce the train of symptoms described. Within the last two weeks I have seen two men who were bitten on the hand by flies. In both instances the men saw the flies and knocked them off. The bite was painful, and the hands swelled and remained swollen and painful for several days. In one of them the fore arm swelled. There was ulceration but no gangreen at the bite.

One was relieved by an ointment of the bichloride of mercury, and the other by a combination of carbolic acid, camphor and olive oil. The fly that bit these men is very common, and is at present a great pest to cattle. People are frequently bitten by them, but with no such result as in the foregoing cases; therefore, I conclude that the fly must have carried some other poison to the wound besides its own.

